# *In Situ* Immune Response in Human Chromoblastomycosis – A Possible Role for Regulatory and Th17 T Cells

**DOI:** 10.1371/journal.pntd.0003162

**Published:** 2014-09-18

**Authors:** Aline Alves de Lima Silva, Paulo Ricardo Criado, Ricardo Spina Nunes, Wellington Luiz Ferreira da Silva, Luciane Kanashiro-Galo, Maria Irma Seixas Duarte, Mirian N. Sotto, Carla Pagliari

**Affiliations:** 1 Laboratório da Disciplina de Patologia de Moléstias Transmissíveis/Departamento de Patologia, Faculdade de Medicina da Universidade de São Paulo, São Paulo, Brazil; 2 Laboratório de Dermatopatologia/Departamento de Dermatologia, Faculdade de Medicina da Universidade de São Paulo, São Paulo, Brazil; Fundação Oswaldo Cruz, Brazil

## Abstract

**Background:**

Chromoblastomycosis is a chronic fungal infection that affects skin and subcutaneous tissue. Lesions can be classified in tumorous, verrucous, cicatricial and plaque type. The cellular immune response in the severe form of the disease seems to correlate with a Th2 pattern of cytokines. The humoral immune response also seems to play a role. We intended to explore the populations of regulatory T cells and the Th17 pattern.

**Methodology:**

Twenty-three biopsies of verrucous form were obtained from patients with clinical, culture and histopathological diagnostic of chromoblastomycosis, without treatment. It was performed an immunohistochemistry method to detect Foxp3, CD25, TGF-β, IL-6, IL-17 and IL-23.

**Principal findings:**

IL-17 was the only cytokine with high expression in CBM when compared to normal skin. The expression of Treg cells, TGF- β, IL-6 and IL-23 were similar to normal skin.

**Conclusions/Significance:**

The constitution of a local immune response with high expression of IL-17 and low expression of other cytokines could be at least in part, an attempt to help the immune system against fungal infection. On the other hand, high levels of local immune response mediated by Th17 profile could overcome the role of Treg cells. The inefficient immunomodulation as a consequence of the unbalance by Treg/Th17 cells seems to corroborate with the less effective immune response against fungi.

## Author Summary

Chromoblastomycosis (CBM) is a chronic infection that affects skin and subcutaneous tissue, caused by some fungi which have a brownish color due to the presence of melanin pigments. The most frequent lesions are of verrucous type. Here we describe the participation of regulatory T cells and cells with the Th17 pattern of cytokines. High levels of Th17 cells participate in chronic inflammatory conditions, once at least in part they could improve the immune response and act in concomitance to the Th1 and Th2 patterns. Our results indicate a predominance of the Th17 pattern over Treg cells in verrucous lesions. We speculate that the local immune imbalance in CBM lesions characterized by exacerbated Th17 response, probably by suppressing the Treg response, is not effective for total fungal elimination. Even after long-period treatments, most patients has no absolute cure and often there is recurrence of the lesions. We believe that our study could contribute to the understanding of the immunopathogenesis of CBM and in such a way, presents some aspects to new possible therapies.

## Introduction

Chromoblastomycosis (CBM) is a chronic granulomatous fungal infection that affects skin and subcutaneous tissue, especially the lower limbs. It is a cosmopolitan disease, but classically it is found in tropical and subtropical regions [1].

The causative agents of CBM are dimorphic fungi [2], [3] which have a brownish color due to the presence of melanin pigments in their cell wall and the spherical morphology that allow easy histological and mycological identification [4]–[8].

The infection occurs following traumatic inoculation of conidia or mycelial fragments from dematiaceous fungus [9]. The most frequently isolated species are *Fonsecae pedrosoi*, *Phialophora verrucosa*, *Cladophialophora carrionii* and eventually *Rhinocladiella aquaspersa* [10].

Clinically, the lesions can be classified in two instances: one that takes into account the appearance (tumorous, verrucous, cicatricial and plaque type) and the other, considering the gravity (mild, moderate or severe, according to the number and size of lesions) [11], [12], [13].

In general, CBM begins with the eruption of papules or nodules that develop slowly into warts. Regularly lesions appear in the lower limbs, knees and hands. There are reports of involvement of the face, chest, buttocks and other areas; dissemination via the lymphatic system is possible but infrequent [12].

The mechanisms of host defense in CBM are not fully elucidated. It is known that the immune response against the CBM is primarily cellular, where the process is ordered by phagocytic macrophages and humoral response also plays a role. Langerhans cells and Factor XIIIa+ dermal dendrocytes appear to be involved to a lesser extent in phagocytosis and antigen presentation against *F. pedrosoi* [6], [14]–[16].

Some studies have investigated the polarity of CBM and demonstrated, both experimentally and *in situ*, that the severe form of the disease, or warty lesions, correlates with a Th2 pattern of immune response by presenting the production of IL-10, high fungal burden, and also TNF-α. The average form is related to the Th1 profile, with high production of IFN-γ, low levels of IL-10, scarce number of fungi and relates to the better granulomatous immune response, resulting in less severe injuries (plaque type lesion) [17], [18].

Several studies have been conducted to better understand the Th1/Th2 paradigm of immune response or, in some diseases, the presence of both patterns of cytokines in the host. It is known that there is a subset of T lymphocytes that can modulate the immune response against pathogens, self-antigens and allergens which is also a constituent of immunological tolerance, called regulatory T cells (Treg). They are characterized by the expression of high levels of CD25 (α chain of the IL-2) whose function depends directly on the transcription factor Foxp3 [18]–[20].

A cytokine of high value for the studies of the immune response is TGF-β, since it is involved in the healing process in order to minimize tissue damage [21] and suppresses CD8^+^ cells [22], transform CD4^+^CD25^−^ cells into CD4^+^CD25^+^ [23], induce naive T cells to differentiate into Foxp3+ [24] and still participates in differentiation of CD4 T cells in Th17 cells [25].

High concentrations of TGF-β added to the absence of pro-inflammatory cytokines direct the immune response to the development of regulatory T cells. Similarly, low concentrations of TGF-β associated with pro-inflammatory cytokines such as IL-1 β, IL-6, IL-21 and IL-23 promote expression of the IL-23 receptor (IL-23R), factor that allows the differentiation of CD4 T cells in Th17 [26], [27].

The Th17 lineage is a subpopulation of CD4^+^ T cells characterized by the secretion of IL-17 that seems to reinforce the protection of the host when the immune profiles of Th1 and Th2 cells are not totally effective against intracellular pathogens. Target of scientific spotlight, these cells appear to be protagonists in chronic inflammatory conditions, such as psoriasis [28], [29].

The study of both cell lines was performed by Pagliari *et al.* [30] in specimens from patients with paracoccidioidomycosis skin and mucous membranes lesions. The analysis revealed the involvement of both cellular profiles, identifying both the presence of immunoregulatory mechanism as the strengthening of effector T cells expressing IL-17.

Taking into account that, although the Th17 cells and regulatory T have similar ontogeny but distinct roles in the generation and control of infections [31], exploring their relationship could improve the understanding of the immunopathology of chromoblastomycosis and contribute to the most effective therapeutic strategies against the disease.

## Materials and Methods

### Biopsies

Twenty-three biopsies from skin lesions were kindly provided by Dermatopathology Laboratory, Division of Clinical Dermatology, Hospital das Clinicas, Faculty of Medicine, University of São Paulo, obtained from patients with clinical, culture and histological diagnostic of chromoblastomycosis by *F. pedrosoi* (86.96% males, mean age 61 years old, SD 15.28). The control group was constituted by ten specimens of normal skin, free of infectious or inflammatory activity at the time of surgery.

In addition to the skin control group without inflammatory activity (n = 10), it was also used a group of twenty skin lesions of paracoccidioidomycosis (PCM). This disease is caused by the dimorphic fungus *Paracoccidioides brasiliensis* and the host immune response against this fungus shares some similarities with chromoblastomycosis. Moreover, some markers of immune response proposed in this work have been described and discussed in PCM.

The use of the material that constituted the casuistic was approved by the ethics committee of Hospital das Clínicas da Faculdade de Medicina da Universidade de São Paulo, under the number 0317/11.

### Immunohistochemistry

It was performed a streptavidin-biotin peroxidase method. The specimens were deparaffinized and hydrated in ethanol, the antigens were retrieved in TRIS/EDTA buffer pH 9.0 for 20 minutes at 95°C. The primary antibodies anti-Foxp3 (clone 236A/E7), anti-CD25 (clone 4C9), anti-TGF-β (clone TGFB17), IL-6 (clone 10C12), IL-17 (clone IL17A) and IL-23 (clone HLT2736) were diluted in 1% bovine albumin solution and incubated over-night at 4°C. Following, it was applied the second antibody and Streptavidin-peroxidase complex. 3,3-diaminobenzidine tetrahydroxychloride was used as chromogen and the slides were counterstained with hematoxylin. All reactions were performed with positive and negative controls. The second ones were constituted by the use of isotype controls and the omission of the primary antibody.

### Quantitative analysis

Cells were quantified by counting the number of immunolabeled cells in nine randomized high-power fields for each specimen with an ×10 ocular lens with a square grid area of 0.0625 mm^2^. The number of positive cells was statistically analyzed with the Mann-Whitney test with the level of significance set at 95%.

## Results

**Figure 1:**
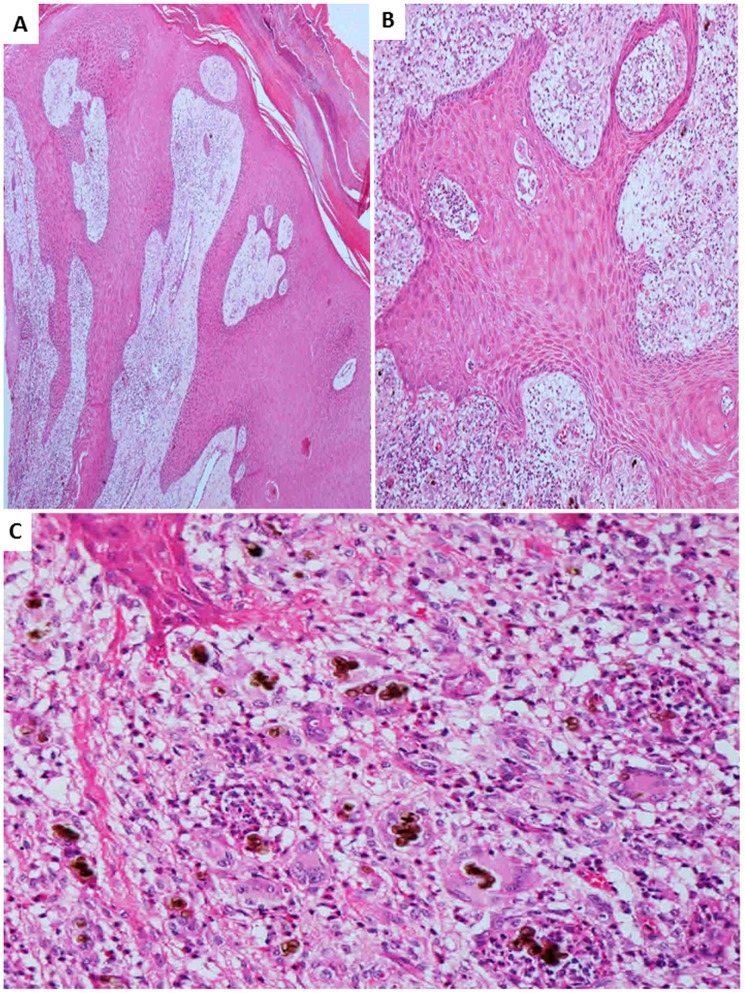
Chromoblastomycosis – Histopathology of skin lesions. A: epidermis presenting pseudocarcinomatous hyperplasia and acantosis (×40); B: presence of acantosis and intraepidermal microabscess (×100); C: dermal inflammatory infiltrate characterized by granulomatous response with giant cells, high number of fungal cells, eosinophils, macrophages and lymphocytes (×200) (hematoxylin-eosin).

The group of CBM specimens consisted of lesions with a verrucous aspect. The histopathological analysis evidenced epidermal changes as hyperkeratosis, irregular acanthosis and microabscesses. The dermis was constituted by suppurative granulomas, inflammatory infiltrate consisting of giant cells, epithelioid cells, macrophages, lymphocytes, plasma cells, and eosinophils. The semi-quantitative analysis of parasitism ranged from moderate to intense. Eventually it was possible to identify ulceration of the skin, pseudocarcinomatous hyperplasia and fibrosis (Fig. 1).

**Figure 2:**
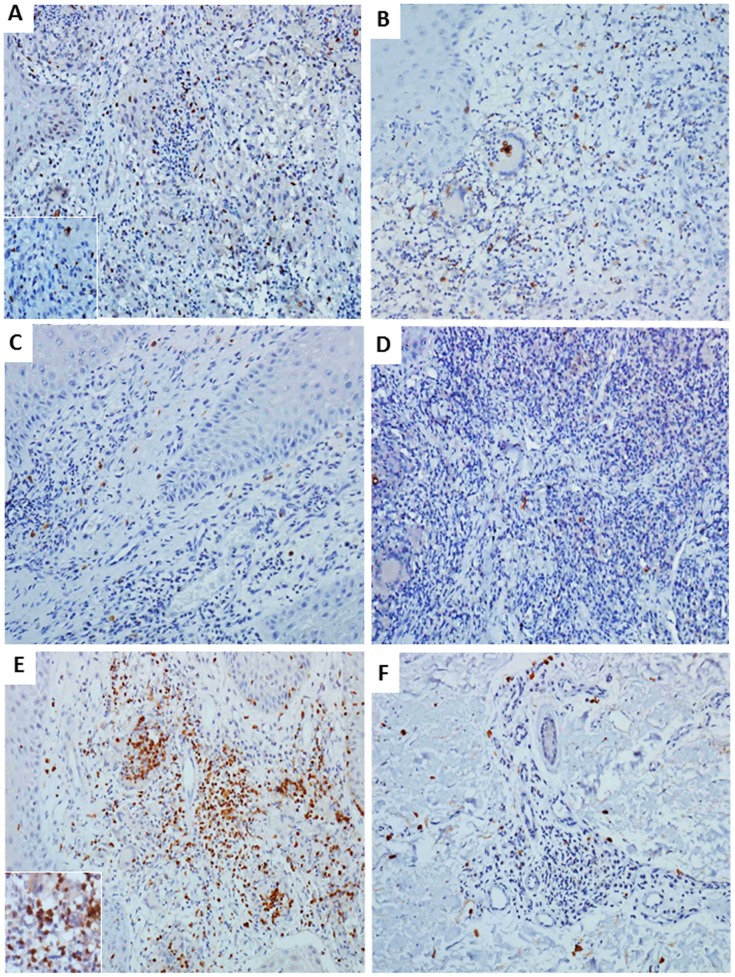
Immunohistochemistry of representative chromoblastomycosis skin lesion. A: Nuclear expression of Foxp3 in regulatory T cells. B: expression of CD25 in lymphocytes of the inflammatory infiltrate. C: Low number of cells expressing TGF-β. D: Scarce presence of IL-6. E: Intense number of T cells with expression of IL-17 in the inflammatory infiltrate. F: Low number of cells expressing IL-23 distributed in the lesion. Streptavidin-biotin peroxidase, immunostaining in brown (×200 and inset ×400).

**Figure 3:**
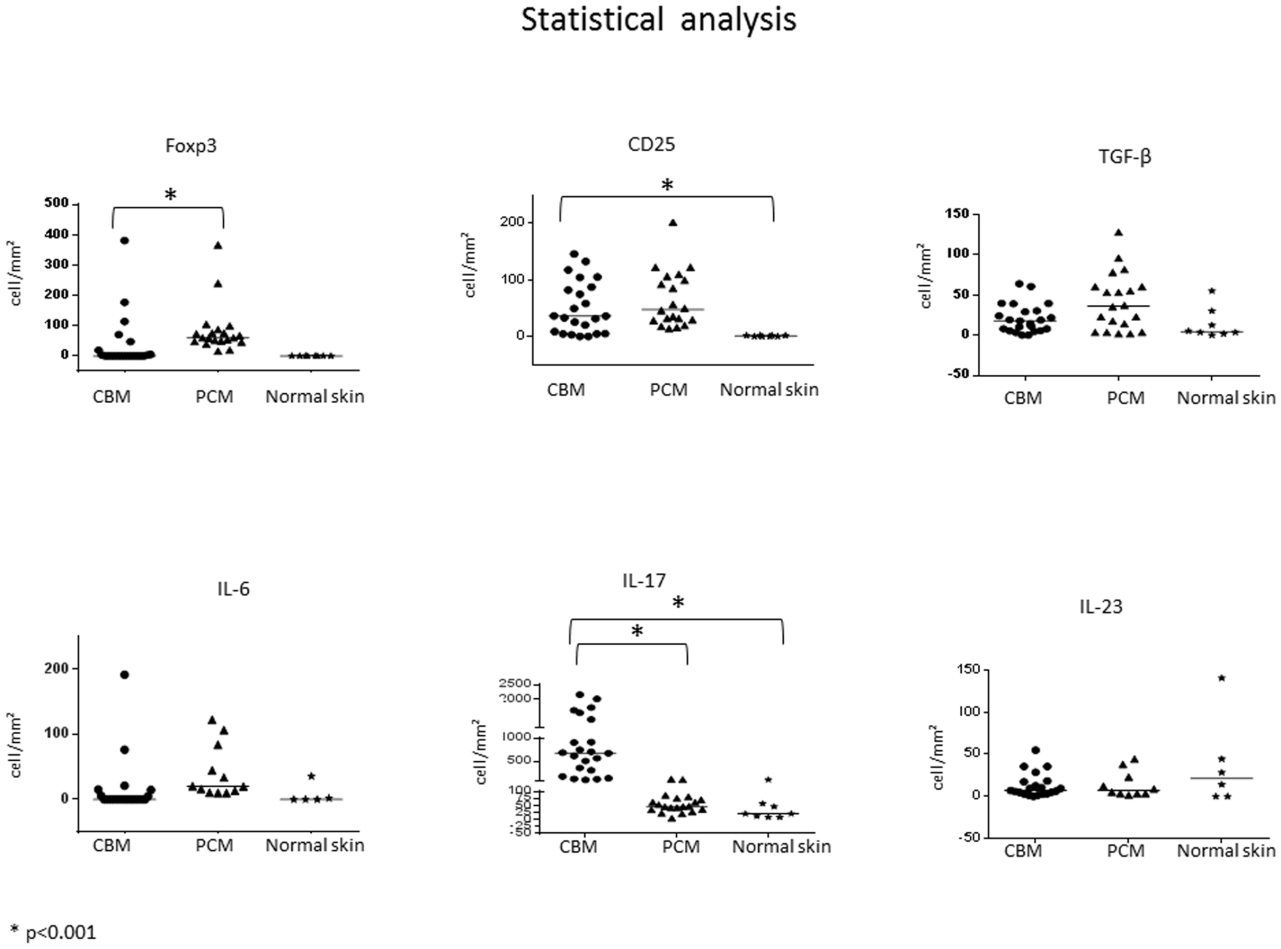
Comparative analysis among chromoblastomycosis, paracoccidioidomycosis and normal skin groups. Distribution of number of cells/mm^2^ expressing Foxp3, CD25, TGF-β, IL-6, IL-23 and IL-17. Mann-Whitney test, * p<0.05.

The immunohistochemical method allowed observing cells expressing Foxp3 and CD25, both in the inflammatory infiltrate and around granulomas. The expression of TGF-β was present in mononuclear cells of the inflammatory infiltrate. There was a discrete expression of IL-6. The expression of IL-17 was visualized in mononuclear and polymorphonuclear cells, mainly in granulomatous areas. Cells expressing IL-23 were present in the inflammatory infiltrates (Fig. 2 and 3).

The group of PCM was characterized by the presence of all markers, expressed in mononuclear cells in the inflammatory infiltrate similar to the CBM group.

The control group of normal skin also presented the markers. However, the expression was low or even absent in some cases.

Cells expressing IL-23 were detected in 66% of the normal skin specimens. Those cells were observed only in the dermis.

The statistical analysis of cells expressing Foxp3 evidenced a decreased number of such cells in CBM group when compared to PCM group (p<0.001) and similar number when compared to normal skin.

The expression of CD25 was similar between CBM and PCM groups, however CBM group presented a statistically significant higher number of positive cells when compared to normal skin (p<0.001).

The number of cells expressing TGF-β was similar among the three groups (p = 0.05), the same to IL-6 and IL-23 (p>0.05).

The three groups presented cells expressing IL-17. However, CBM group had an increased number when compared to the two other (p<0.001).

The quantitative analysis can be visualized in figure 3 and table S1.

## Discussion

The study of CBM skin lesions is important because these injuries can reflect the immune status of the patient. The cutaneous tissue is often responsible for the initiation of immune cascade and, in the case of chromoblastomycosis, it is the structure to which the fungus has tropism.

Studies concerning immune response in CBM show that verrucous lesions present the mycotic granuloma formation with a pattern of Th2 response consistent with worse response against fungus. On the contrary, lesions of plaque type are characterized by a Th1 pattern of cytokines and therefore a better immune response, according to Minotto (unpublished data) and D'ávila [17]. However, there are no reports studying the immune dynamics of the lesions or inferences about what it takes to develop such responses.

We evidenced the predominance of cells expressing IL-17 and the presence of TGF- β and IL-23, although in low number. According to the literature published, this pattern of cytokines characterizes the Th17 immune response. The concomitant presence of a Th17 pattern could represent at first, an attempt of the *in situ* immune system to restrain the fungal infection.

With respect to the presence of regulatory T cells, although to a lesser extent when compared to cells expressing IL-17, we were also able to verify considerable number of Foxp3+ cells (40% of cases) and CD25+ cells (90%).

According to Melo and Carvalho [32], regulatory T cells have a key role in maintaining tolerance and regulation of the immune response. In the same way, in a work of Weaver [28] it is discussed that Th17 cells appear to be the translation of adaptability of the immune system favoring the protection of the host, so that the elucidation of the characteristics of both groups of cells, i.e. Treg and Th17 cells, could not only identify the mechanisms of invading organisms, but also to assist in the development of more effective therapies for numerous diseases.

Treg cells are characterized by a CD4+CD25+ phenotype [33], however the CD25 molecule is expressed by others populations of cells, such as others T cells and B lymphocytes or activated macrophages. Studies demonstrated that the gene Foxp3 has a nuclear expression in Treg cells and therefore a reliable marker of this cell line [34].

As already seen, the cytokine TGF-β plays a dichotomy role and mediates the targeting of Treg and Th17 responses. This selection between both profiles seems to depend on the levels of cells and the cytokines present in the microenvironment. High levels of TGF- β and low expression of pro-inflammatory cytokines favor the regulatory T-profile. On the contrary, low levels of TGF-β associated to significant presence of pro-inflammatory cytokines promote the synthesis of IL-23, development and maintenance of Th17 lymphocytes [35]–[41].

The cytokine IL-17 has received a considerable attention in many contexts since its discovery in 1993 [42]. The cytokine IL-23 promotes the secretion of IL-17 produced mainly by CD4+ T cells [43], [44].

According to many studies, high levels of Th17 cells are protagonists in chronic inflammatory conditions, once this cell population, at least in part, could improve the immune response and act in concomitance to the Th1 and Th2 patterns of response [28]. The performance of this cell line is characterized by secretion of IL-6, IL-17, IL-22 and TNF-α [45].

In the same context of investigation we consider patients affected by paracoccidioidomycosis (PCM), a systemic mycosis similar in some aspects to CBM. The disease follows inhalation of conidia of *Paracoccidioides brasiliensis*, a dimorphic fungus [46], and the primary focus are the lungs. It is described the cutaneous involvement at about 30% of cases. Unlike CBM, the lympho hematogenous spread of fungi is more frequent [47].

The cellular immune response in PCM is mainly mediated by macrophages and CD4+T cells [48], [49]. The Th1 profile of cytokines is considered the most important and some studies have also demonstrated the role of Treg cells and the profile of Th17 cytokines [30].

Considering previous investigations on the role of Treg cells in PCM, our results could suggest that this cell population not only have the capacity to interfere in the efficient immune response against fungi in chromoblastomycosis, but also benefit the host, by being able to reduce the tissue damage that follows a local immune response [30], [50].

In a recent study, the authors discussed the interaction of cells producing IL-17 and Treg cells and the homeostasis of the intestinal mucosal tissue [51]. The unregulated interaction of pro-inflammatory activity of IL-17 with pathogens seems to change the balance between regulatory and effector response predisposing the individual to the chronicity of the disease. It is noteworthy that even being subjected to long-period treatments, most patients affected by CBM has no absolute cure and often there is recurrence of the lesions. Thus, the unbalance between the populations of Treg/Th17 cells seems to restrain the effective immune response against the fungus.

Finally, it was interesting the similar number of cells expressing TGF-β, IL-6 and IL-23 when we compared the groups of lesions and normal skin. We expected that both CBM and PCM specimens presented more cells expressing those markers. We speculate that, at least in part, the presence of such cytokines in normal skin could be produced by the components of skin immune system [52].

Interestingly, in a previous work, Esterre *et al.* (1994) observed an overexpression of TGF-beta mainly at the periphery of the granulomas in areas of fibrosis [53].

In this work, we could suggest that the low number of cells with TGF-beta in lesions of CBM, with no difference from normal skin, could be explained by the randomized counting of positive cells throughout the dermis and not specifically in areas of fibrosis where such cells were also observed.

We suggest that our study could contribute to the understanding of the immunopathogenesis of chromoblastomycosis and in such a way, presents some aspects that could assist in the possibility of new therapies to modulate the immune system to the most effective immune profile of patients.

## Supporting Information

Table S1 **Quantitative analysis: Numerical results of the markers studied.** Results are given as mean ± standard deviation.
(TIF)
