# Supplementary figures and images for: In Situ Immune Response in Human Chromoblastomycosis – A Possible Role for Regulatory and Th17 T Cells
Source: PLoS Negl Trop Dis. 2014 Sep 18;8(9):e3162. doi: 10.1371/journal.pntd.0003162 (PMC4169370; doi:10.1371/journal.pntd.0003162)

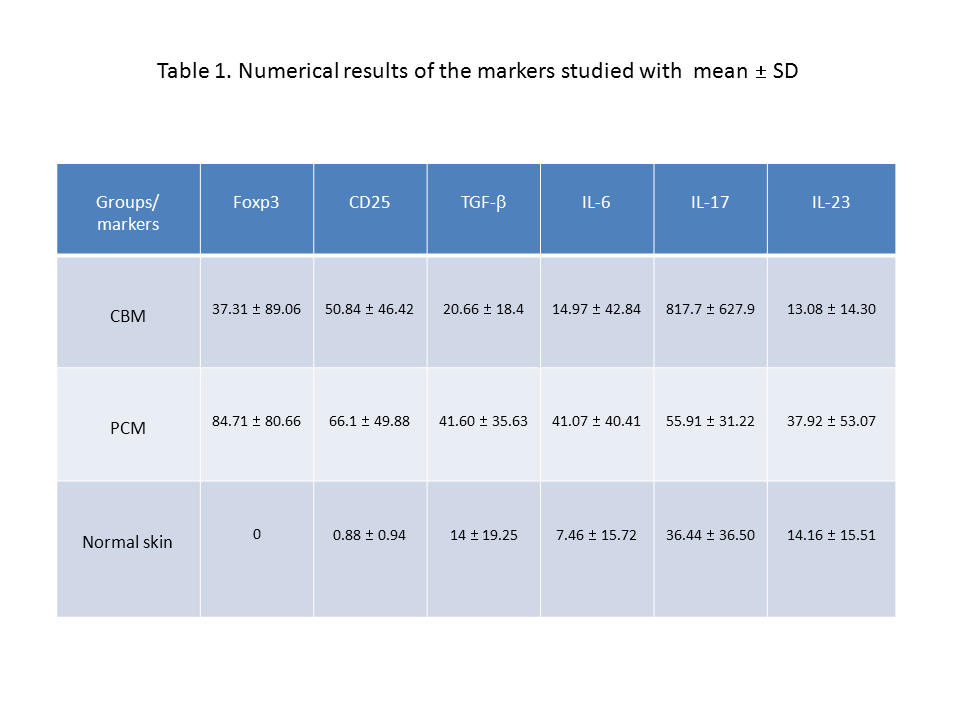

Supplement: Table S1 — Quantitative analysis: Numerical results of the markers studied. Results are given as mean ± standard deviation. (TIF) [file pntd.0003162.s001.tif]
